# Increased Risk of Glaucoma in Patients with Rosacea: A Nationwide Population-Based Cohort Study

**DOI:** 10.3390/jcm12113759

**Published:** 2023-05-30

**Authors:** Kyunghee Chae, Suyeon Kim, Sukil Kim, Yu Ri Woo

**Affiliations:** 1 Department of Preventive Medicine, College of Medicine, The Catholic University of Korea, Seoul 06591, Republic of Korea; 2 Department of Dermatology, Incheon St. Mary’s Hospital, College of Medicine, The Catholic University of Korea, Seoul 06591, Republic of Korea

**Keywords:** rosacea, glaucoma, risk, ocular, nationwide

## Abstract

Rosacea is a chronic inflammatory skin disorder associated with various ocular manifestations. However, little is known about the association between rosacea and glaucoma. This study aimed to determine the risk of glaucoma in patients with rosacea. This nationwide population-based retrospective cohort study enrolled 1056 individuals with rosacea and 10,440 age- and sex-matched controls without rosacea from the Korean National Health Insurance System (NHIS) database from 2002 to 2015. The incidence rate of glaucoma was 1215.4 per 100,000 person-years (PYs) in patients with rosacea and 741.3 per 100,000 PYs in patients without rosacea. A significantly higher cumulative incidence probability of glaucoma was observed in patients with rosacea than in non-rosacea controls (*p* = 0.0004). Rosacea was associated with an increased risk of developing glaucoma (adjusted hazard ratio [aHR], 1.659; 95% confidence interval [CI], 1.245–2.211) compared to those without rosacea. In subgroup analysis, increased risk of glaucoma was observed in patients with rosacea younger than 50 years (aHR, 1.943; 95% CI, 1.305–2.893), females (aHR, 1.871; 95% CI, 1.324–2.644), and patients with hypertension (aHR, 1.561; 95% CI, 1.037–2.351) compared to those without rosacea. Rosacea is associated with an increased risk of developing glaucoma. Proper screening for glaucoma should be conducted in rosacea patients younger than 50 years, females, and patients with hypertension to better control the disease and prevent vision loss from glaucoma.

## 1. Introduction

Rosacea is a chronic inflammatory skin condition that primarily affects the central part of the face. Clinically, patients with rosacea suffer from recurrent episodes of flushing, papules, pustules, erythema, and telangiectasia on the face. Rosacea primarily affects patients in the third to fifth decades of life but can be present at any age [1].

The exact cause of rosacea is not fully understood, but it is thought to involve a combination of the genetic, environmental, innate and adaptive immune system, and microbial imbalances [2]. In addition, neurovascular dysfunction is thought to play a crucial role in the pathogenesis of rosacea. With rosacea, it is believed that an overactive nervous system may cause blood vessels in the face to dilate too easily, resulting in flushing, edema, and erythema. The dilation of blood vessels is thought to be caused by the release of neuropeptides, such as the calcitonin gene-related peptide (CGRP) and substance P [3,4]. Chronic neurovascular dysfunction can also lead to the development of telangiectasia, which is a common feature of rosacea. Over time, the abnormal dilation and constriction of blood vessels can lead to structural changes in the blood vessels, which can make them more prone to dilation and further exacerbate the symptoms of rosacea.

Rosacea can be classified into four clinical subtypes, which include erythematotelangiectatic, papulopustular, phymatous types, and ocular rosacea [5]. Patients with rosacea exhibit multiple overlaps of these subtypes. Rosacea is currently diagnosed by a new diagnostic system updated in 2017 [6]. The diagnostic features of rosacea are fixed centro-facial erythema in a characteristic pattern that may periodically intensify [6]. In addition, the presence of two or more major features, including flushing, papules and pustules, telangiectasia, and ocular manifestations, can be considered diagnostic features of rosacea [6]. Scleritis/sclerokeratitis, spade-shaped infiltration in the cornea, lid margin telangiectasia, and conjunctival injection are important manifestations of ocular rosacea [6].

The incidence of ocular rosacea varies by studies, ranging from 6 to 72%, and this variation is observed between dermatologic and ophthalmologic studies [7,8]. Ocular rosacea can be overlooked by physicians [9] because the severity of clinical symptoms and signs of ocular rosacea do not usually correlate with those of facial rosacea [10]. Nevertheless, ocular rosacea is often comorbid in patients with facial rosacea, and some studies have confirmed the possible association of various ophthalmologic disorders other than manifestations of ocular rosacea in patients with rosacea [11,12].

Glaucoma is one of the most common disorders among various ophthalmic disorders, and it is a leading cause of blindness worldwide [13]. Open-angle glaucoma is the most common type, accounting for about more than 80% of all cases [13]. Other types of glaucoma, such as angle-closure glaucoma and secondary glaucoma, are less common [13]. The exact mechanisms of glaucoma development are not fully identified. However, several factors, including genetic factors, increased intraocular pressure (IOP), older age, optic nerve damage, oxidative stress, endoplasmic reticulum (ER) stress, and neurovascular inflammation, have been identified as potential contributors to the disease [13,14,15,16]. Abnormalities in the immune system, oxidative stress, ER stress, and abnormal neurovascular condition are also considered to be crucial factors in rosacea pathogenesis [17,18]. So, it is necessary to confirm the association between rosacea and glaucoma. As glaucoma can lead to irreversible blindness, early diagnosis and identification of risk factors are necessary to prevent further vision loss.

Little is known about the association between new-onset glaucoma and rosacea. Therefore, we aimed to evaluate the incidence rate of glaucoma in patients with rosacea using a nationwide population-based dataset. In addition, we determined whether rosacea is associated with an increased risk of new-onset glaucoma and aided in identifying the possible risk factors associated with developing glaucoma in patients with rosacea.

## 2. Methods

### 2.1. Data Source and Design

We used the National Health Insurance Service—National Sample Cohort (NHIS-NSC) from 2002 to 2015 in South Korea [19]. The NHIS is a government-operated mandatory social health insurance program that contains all claims data for approximately 50 million South Koreans. The NHIS-NSC is a population-based cohort that comprises a mixture of inpatients and outpatient claims by the NHIS and includes 2.2% of the total Korean population, selected through a systematic stratified random sampling method [19].

**Figure 1:**
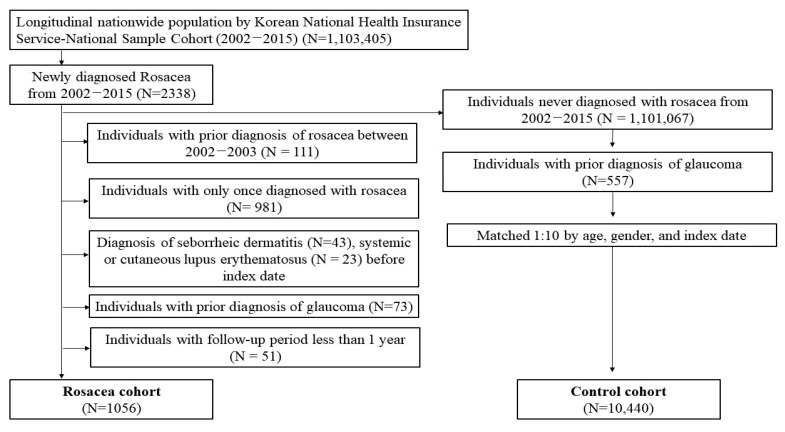
Study flowchart.

We identified 2338 patients that were newly diagnosed with rosacea from 2002 to 2015 based on the International Classification of Diseases (ICD)-10 code L71 (Figure 1). The subjects were those diagnosed with rosacea before enrollment (N = 111), and those with a follow-up period of less than one year were excluded (N = 51). As we defined rosacea patients as those who claimed two or more primary diagnoses of rosacea, we excluded patients with only one such diagnosis (N = 981) to increase the diagnostic validity. We also excluded the patients with rosacea and recorded seborrheic dermatitis (N = 43) or systemic or cutaneous lupus erythematosus (N = 23). Individuals with a prior diagnosis of glaucoma were excluded (N = 73). Finally, 1056 rosacea patients were identified as eligible for inclusion. The controls were selected randomly from age, sex, and index-date-matched individuals without rosacea. Among them, we excluded individuals with a prior diagnosis of glaucoma (N = 557). The patients with rosacea and the controls were followed up until diagnosis of glaucoma or 31 December 2015. The study protocol was approved by the Institutional Review Board (IRB) of the Catholic University of Korea (IRB approval number XC19REDI0064).

### 2.2. Definitions of Glaucoma and Comorbidities

Glaucoma was defined as a primary diagnosis with an ICD-10 code of H40-H42 more than twice during the observation period. To only include cases of developing glaucoma, we only included those diagnosed with glaucoma at least six months after rosacea diagnosis.

Baseline comorbidities were evaluated based on the ICD-10 codes of each comorbidity only when patients were diagnosed with those comorbidities before the diagnosis of rosacea. Hypertension was defined as having at least two claims as a primary diagnosis of ICD-10 codes I10-I13. Dyslipidemia was defined as having at least two claims of ICD-10 code E78. Diabetes mellitus was defined as having at least two claims of ICD-10 codes E10-E11. 

### 2.3. Statistical Analysis

Data are presented as mean ± standard deviation (SD), geometric means (95% confidence interval [CI]), or percentages. Differences between groups were evaluated by Student’s *t*-test for continuous variables or a chi-square test for categorical variables. The incidence of glaucoma was calculated by dividing the total number of incident cases by the follow-up period (person-year [PY]) and is presented as the number of cases per 1000 PYs. A Cox proportional hazard regression analysis was performed to examine the risk of glaucoma in patients with rosacea. To control for confounding factors, we used the models adjusted for age, sex, hypertension, diabetes mellitus, dyslipidemia, and obesity. We performed subsequent subgroup analyses to clarify the risk of glaucoma in terms of sex, age, diabetes mellitus, dyslipidemia, and hypertension. The sensitivity test was conducted to exclude the cases diagnosed with glaucoma followed by rosacea within 1 and 2 years and to calculate the association. Those sensitivity analyses were conducted to examine whether the main findings were robust. Statistical analysis was performed using SAS version 9.4 (SAS Institute Inc., Cary, NC, USA), and a *p* < 0.05 was considered statistically significant.

## 3. Results

### 3.1. Baseline Characteristics

Table 1 summarizes the demographic characteristics of the subjects. We identified 11,496 subjects, among whom 1056 were diagnosed with rosacea and 10,440 were not. Among rosacea patients, the proportion of females (63.06%) was higher than males (36.94%). The mean (SD) age of rosacea patients was 47.02 (15.32) years, and the mean (SD) age of non-rosacea subjects was 46.75 (14.99) years. Among the 1056 patients with rosacea, more patients with rosacea showed comorbid conditions, such as dyslipidemia and diabetes mellitus, than subjects without rosacea.

### 3.2. Incidence and Risk of Glaucoma in Rosacea

**Figure 2:**
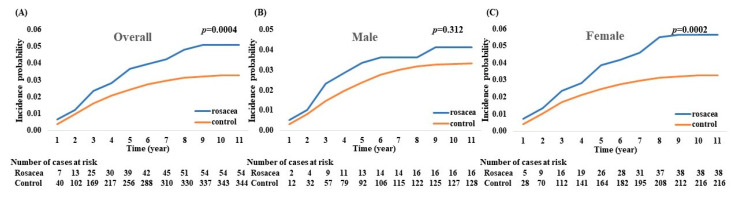
Cumulative incidence of glaucoma in patients with rosacea and the non-rosacea control. (**A**) Overall incidence of glaucoma in rosacea and the non-rosacea control. (**B**) Incidence of glaucoma among males. (**C**) Incidence of glaucoma among females.

During a mean follow-up of 5.23 years, there were 398 cases of glaucoma (3.46%) in the entire cohort. A significantly higher cumulative incidence probability of glaucoma was observed in patients with rosacea than in the non-rosacea controls (*p* = 0.0004) (Figure 2). When we stratified the incidence probability of glaucoma by sex, female patients with rosacea showed a significantly higher cumulative incidence probability of glaucoma than the female non-rosacea controls (*p* = 0.0002).

To further identify the risk of new-onset glaucoma in rosacea patients, we conducted a Cox proportional hazard regression analysis and found an increased risk of developing glaucoma among rosacea patients compared to non-rosacea controls in the non-adjusted model (crude hazard ratio [HR] = 1.663; 95% CI: 1.249–2.216, Table 2). This risk remained significant in the fully adjusted model (aHR = 1.659; 95% CI: 1.245–2.211), which adjusted for age, sex, dyslipidemia, hypertension, and diabetes mellitus.

### 3.3. Subgroup Analysis for Risk of Glaucoma in Rosacea

Subgroup analyses were conducted in patients with rosacea adjusted by age, sex, diabetes mellitus, hypertension, and dyslipidemia. The risk of developing glaucoma was higher in rosacea patients younger than 50 (aHR, 1.943; 95% CI, 1.305–2.893) and in female patients with rosacea (aHR, 1.871; 95% CI, 1.324–2.644) than in the non-rosacea controls. Among hypertensive patients, the risk of glaucoma was increased in patients with rosacea compared to the non-rosacea controls (aHR, 1.561; 95% CI, 1.037–2.351; Table 3).

### 3.4. Sensitivity Analysis

To perform a sensitivity analysis, selective exclusion of patients with rosacea and the controls who were enrolled in our study and subsequently developed glaucoma within one year (aHR, 1.679; 95% CI, 1.285–2.411) or two years (aHR, 1.675; 95% CI, 1.297–2.402) after diagnosis of rosacea resulted in consistent findings, which revealed that developing glaucoma risk was significantly higher among patients with rosacea than in the controls (Table 4).

## 4. Discussion

We found that the cumulative incidence of glaucoma was significantly higher in patients with rosacea than those without rosacea using the nationwide Korean registry database. The risk of newly diagnosed glaucoma increases in patients with rosacea compared to those without rosacea after adjusting for possible confounding factors.

The risk of newly diagnosed glaucoma in rosacea has not been studied well. Previously, a possible association between rosacea and glaucoma (adjusted odds ratio, 1.93; 95% CI, 1.70–2.20) was identified by a conditional logistic regression analysis using data from multiple centers [8]. However, the association between rosacea and newly diagnosed glaucoma has not been investigated previously. The present nationwide population-based study determined the increased risk of newly diagnosed glaucoma in patients with rosacea.

Unlike other dermatological disorders, with rosacea, various ophthalmic conditions can be observed in the form of ocular rosacea. Although ocular rosacea can also be diagnosed without skin manifestations, patients with cutaneous rosacea accompanied ocular rosacea with a high probability [20]. Ocular rosacea is highlighted by scleritis/sclerokeratitis, spade-shaped infiltration in the cornea, lid margin telangiectasia, and intra-palpebral conjunctival injection [6]. Manifestations of ocular rosacea are mainly associated with symptoms of conjunctiva, cornea, and sclera, including burning, stinging, light sensitivity, and foreign object sensation [6]. In addition, non-specific ocular signs of ocular rosacea include dysfunction of the meibomian gland, conjunctivitis, and cylindrical collarette accumulation at the base of the lashes [6,21]. As glaucoma is not an ocular disorder of the conjunctiva, cornea, or sclera, which are commonly involved in ocular rosacea, we suppose that glaucoma in rosacea patients could be considered as a kind of comorbidity of rosacea rather than a form of ocular rosacea.

The mechanisms underlying glaucoma in rosacea are unclear. As angiogenesis and neurogenic inflammation are crucial both in rosacea and glaucoma, we suppose that the shared role of oxidative stress, ER stress, and vascular endothelial growth factors (VEGFs) impact this shared etiology. The increased expression of the VEGF and its receptors is observed in the lesional biopsy specimens or sera of rosacea patients [22,23,24]. In addition, a recent study found the VEGF polymorphism (e.g., VEGF + 450C/G, rs2010963) is associated with an increased risk of rosacea [25,26]. Various VEGF polymorphisms (e.g., VEGF-460 C/T BstUI, rs699947) are associated with glaucoma in patients with glaucoma [27,28]. The increased expression of VEGF is also observed in the aqueous humor [29] and tear samples of patients with glaucoma compared to healthy controls [30]. Various VEGF polymorphisms, along with increased expression of VEGF in the serum, skin, and ocular fluids of patients with rosacea and glaucoma, may contribute to the possible interaction between these two diseases.

In addition, patients with rosacea showed an increased level of serum total oxidant and advanced oxidation protein products compared to the controls [31]. In addition, elevated levels of oxidative stress were observed in the ocular epithelium of patients with glaucoma compared to the controls, and this increase was associated with neurodegeneration in glaucomatous patients [32,33]. In addition to oxidative stress, ER is an essential organelle in the biosynthesis of lipid membranes, post-translational protein processing, and quality control [34]. Most rosacea triggers, such as ultraviolet irradiation, heat, alcohol consumption, or microbiota, are considered ER stressors [34]. With rosacea, various triggers activate ER signaling by the upregulation of activating transcription factor (ATF) 4, which promotes the inflammatory cascade of rosacea via downstream signaling of toll-like receptor (TLR) 2 or 4 [35]. In addition, increased ER stress is associated with increased VEGF-A expression [35], suggesting the role of ER stress in angiogenesis. Likewise, several studies have reported that excessive ER stress is a crucial promoter of glaucoma by inducing neuronal cell death [36,37]. Recent studies found the upregulation of ATF4 in the trabecular meshwork in glaucoma patients [38], and the persistent activation of ATF4 during chronic ER stress was associated with the development of glaucoma [38]. While further studies are required to confirm this association, the shared etiology can potentially explain the link between these two diseases. 

Our subgroup analysis found that rosacea patients younger than 50 years and female patients were at an increased risk of glaucoma. In general, both rosacea and glaucoma are more common in the elderly population. However, we observed that rosacea patients younger than 50 had a higher risk of developing glaucoma than the healthy controls. As we found that the risk of glaucoma can occur earlier in rosacea patients, rosacea patients should be screened for glaucoma even at a relatively young age. In addition, we found that the risk of developing glaucoma increased in female rosacea patients. While both females and males can develop rosacea, the prevalence of rosacea is more common in females, and females may experience more severe symptoms. Severe rosacea symptoms are often indicative of a heightened inflammatory response, particularly observed in females. This can be attributed to the higher frequency of exposure to trigger factors such as hormonal changes and the use of skincare products among females, which can exacerbate the inflammatory reaction in female rosacea patients. Furthermore, these factors may contribute to the increased risk of glaucoma in female rosacea patients. 

Furthermore, our subgroup analyses revealed that, among hypertensive individuals, patients with rosacea showed an increased risk of developing glaucoma compared to the healthy control. Given that systemic hypertension is a well-known risk factor for both glaucoma and rosacea [39,40], this subgroup should be appropriately controlled.

This study had limitations. First, since we utilized big data from an NHIS data warehouse, there was a potential for information bias, which may have led to a misdiagnosis or misclassification of rosacea and glaucoma. Second, due to the study design, we did not consider the impact of glaucoma severity and exposure to rosacea drugs on the development of glaucoma in patients with rosacea. Further studies considering these points are needed. Third, the subclassification of glaucoma diagnoses according to subtypes was not performed in our study. We acknowledge that relying solely on ICD code-based diagnoses in Korea may not always accurately reflect the underlying cause of glaucoma. The use of gonioscopy or anterior segment optical coherence tomography to assess the angle status was not consistently implemented, which could lead to potential misclassification. Additionally, the measurement of intraocular pressure (IOP) using Goldmann applanation tonometry was not uniformly conducted. Consequently, it becomes challenging to differentiate normal-tension glaucoma and angle-closure glaucoma solely based on the ICD code in Korea. Therefore, we opted to include overall glaucoma based on ICD code-based diagnoses for the purposes of this study. Nevertheless, we suggest that the findings from this study provide insight into the link between rosacea and glaucoma.

In conclusion, our findings suggest that patients with rosacea are at a higher risk of developing new-onset glaucoma. Rosacea is a condition that involves not only skin but also eye. The ocular rosacea, which is often underdiagnosed, requires more attention beyond cutaneous rosacea. In addition to the well-known ophthalmic conditions associated with ocular rosacea, clinicians and rosacea patients should be vigilant about the potential development of glaucoma in rosacea patients. Glaucoma, being a significant ophthalmic disease that can lead to blindness, warrants regular screening in rosacea patients. Early screening for glaucoma is crucial to provide timely intervention, and particular attention should be given to subgroups such as patients younger than 50 years, females, and those with concomitant hypertension. By implementing early screening and intervention for glaucoma, we can effectively identify and manage the comorbid conditions of rosacea, ultimately improving the overall disease management outcomes.

## Figures and Tables

**Table 1:** Demographic characteristics at baseline.

	Rosacea (N = 1056)	Non-Rosacea (N = 10,440)	*p*-Value
Age, years, mean (SD)	47.02 ± 15.32	46.75 ± 14.99	0.61
<20	47 (4.16%)	400 (3.63%)	
20–30	130 (11.51%)	1280 (11.62%)	
30–40	149 (13.2%)	1460 (13.25%)	
40–50	289 (25.6%)	2890 (26.23%)	
50–60	281 (24.89%)	2807 (25.48%)	
60–70	157 (13.91%)	1550 (14.07%)	
>70	76 (6.74%)	630 (5.72%)	
Sex			0.82
Male	417 (36.94%)	4027 (36.56%)	
Female	712 (63.06%)	6990 (63.44%)	
Dyslipidemia, yes	464 (42.11%)	3845 (34.90%)	<0.01
Hypertension, yes	311 (28.22%)	3019 (27.40%)	0.42
Diabetes mellitus, yes	256 (23.23%)	2044 (18.55%)	0.003

Values are means ± standard deviations (SD) or numbers (%). Abbreviations: N, number; SD, standard deviations.

**Table 2:** Incidence rate and risk of glaucoma in patients with rosacea.

	Number of Events	Total Number	PYs	Incidence Rate per 100,000 PYs	Crude HR (95% CI)	Model 1 aHR (95% CI)	Model 2 aHR (95% CI)
Control	345	10,440	46,538.2	741.3	*1 (Reference)*	*1 (Reference)*	*1 (Reference)*
Rosacea	54	1056	4442.6	1215.4	1.663 (1.249–2.216)	1.679 (1.260–2.237)	1.659 (1.245–2.211)

Model 1 was adjusted for age and sex. Model 2 was adjusted for age, sex, diabetes mellitus, hypertension, and dyslipidemia. Abbreviations: CI, confidence interval; HR, hazard ratio; PYs, person-years.

**Table 3:** Subgroup analysis of the risk of new-onset glaucoma among patients with rosacea.

Variables	Group	Glaucoma	
		Crude HR (95% CI)	aHR (95% CI)
Age			
Age < 50	Control	*1 (Reference)*	*1 (Reference)*
	Rosacea	1.984 (1.333–2.953)	1.943 (1.305–2.893)
Age ≥ 50	Control	*1 (Reference)*	*1 (Reference)*
	Rosacea	1.422 (0.937–2.157)	1.420 (0.935–2.155)
*Sex*			
Male	Control	*1 (Reference)*	*1 (Reference)*
	Rosacea	1.306 (0.777–2.196)	1.311 (0.779–2.205)
Female	Control	*1 (Reference)*	*1 (Reference)*
	Rosacea	1.886 (1.336–2.662)	1.871 (1.324–2.644)
*Diabetes mellitus*			
No	Control	*1 (Reference)*	*1 (Reference)*
	Rosacea	1.440 (1.182–1.754)	1.512 (1.241–1.842)
Yes	Control	*1 (Reference)*	*1 (Reference)*
	Rosacea	0.847 (0.481–1.491)	0.844 (0.479–1.486)
*Hypertension*			
No	Control	*1 (Reference)*	*1 (Reference)*
	Rosacea	1.257 (0.708–2.231)	1.446 (0.980–2.134)
Yes	Control	*1 (Reference)*	*1 (Reference)*
	Rosacea	1.440 (0.958–2.136)	1.561 (1.037–2.351)
*Dyslipidemia*			
No	Control	*1 (Reference)*	*1 (Reference)*
	Rosacea	1.398 (1.134–1.724)	1.429 (1.159–1.782)
Yes	Control	*1 (Reference)*	*1 (Reference)*
	Rosacea	1.230 (0.819–1.849)	1.293 (0.859–1.945)

HR was adjusted for age, sex, diabetes mellitus, hypertension, and dyslipidemia. Abbreviations: aHR, adjusted HR; CI, confidence interval; HR, hazard ratio.

**Table 4:** Sensitivity analysis of developing glaucoma among patients with rosacea and the control.

Exclusion of Observation Period			
Hazard Ratio of Glaucoma	Total	1-Year Lag Period	2-Year Lag Period
Control	*1 (Reference)*	*1 (Reference)*	*1 (Reference)*
Rosacea	1.659 (1.245–2.211)	1.679 (1.285–2.411)	1.675 (1.297–2.402)

## Data Availability

Data sharing not applicable.
